# Weight change and incident metabolic syndrome in Iranian men and women; a 3 year follow-up study

**DOI:** 10.1186/1471-2458-9-138

**Published:** 2009-05-13

**Authors:** Azadeh Zabetian, Farzad Hadaegh, Parvin Sarbakhsh, Fereidoun Azizi

**Affiliations:** 1 Prevention of Metabolic Disorders Research Center, Research Institute for Endocrine Sciences, Shaheed Beheshti University of Medical Sciences, Tehran, Iran; 2 Endocrine Research Center, Research Institute for Endocrine Sciences, Shaheed Beheshti University of Medical Sciences, Tehran, Iran

## Abstract

**Background:**

Although the association of weight gain and developing metabolic syndrome (MetS) has been reported in the Western and Asian populations, data on the gender-stratified effects of weight change (including weight loss) on incident MetS and its components in the Middle East Caucasians is still scarce.

**Methods:**

A total of 1431 men and 2036 women aged ≥ 20 years with BMI > 18.5 kg/m^2 ^were followed over 3 years. Multivariate logistic regression analysis was used to estimate the relative risk (RR) of MetS and its components (the Adult Treatment Panel III definition) associated with gender-stratified quintiles of percent weight change. Subjects with MetS at baseline were excluded for analyzing the RR of MetS.

**Results:**

There was 20.4% (95% CI, 19.6–21.2) age-adjusted incident MetS (18.4% male vs. 23.1% women). In men, mild weight gain (WG) predicted high waist circumference (WC) and high triglyceride; moderate WG predicted MetS (RR 2.5, 95% CI 1.4–4.3), high WC and high blood pressure (BP); large WG predicted MetS (RR 3.2, 95% CI 1.8–5.7) and its components, except for high fasting plasma glucose. In women, mild WG predicted MetS (RR 2.5, 95% CI 1.4–4.3), high WC and high BP; moderate WG predicted Mets (RR 4.6, 95% CI 2.7–8.0), high WC and high triglyceride; large WG predicted MetS (RR 6.6, 95% CI 3.8–11.3) and its components except for low HDL-cholesterol. Mild weight loss had protective effect on high WC in both genders and MetS in men (RR 0.5, 95% CI 0.26–0.97, P = 0.04).

**Conclusion:**

Weight change showed different effects on MetS in men and women. In women, mild WG predicted MetS; however, mild weight loss was protective against MetS in men and high WC in both genders.

## Background

The prevalence of overweight and obesity is increasing worldwide [1], and body weight is closely associated with mortality and morbidity [2,3]. Obesity has been known as an important risk factor of cardiovascular disease (CVD) [4] and weight reduction in overweight or obese individuals was known to have beneficial health effects [5]. It has been suggested that weight gain increases risk of developing hyperinsulinemia [6] and insulin resistance is an origin of metabolic syndrome (MetS) which is a strong risk factor of CVD [7]. Therefore, it seems that investigations on the association of weight change and MetS are essential to clarify the relationship between weight change and mortality.

Although self-reported weight gain is proven to be associated with the development of the MetS [8], only a few studies have prospectively assessed the impact of documented weight gain on MetS or its components [6,9]. Furthermore, not much published data is available about the effect of weight loss on MetS and its components [6,10-12].

On the other hand, body weight is a different entity among men and women [13] and the association between body mass index (BMI) and mortality was found to be different for men and women [14]. Therefore, to gain better knowledge about the weight gain epidemic and its adverse effect on MetS parameters, we believe that investigations stratified by gender need to be conducted.

Considering the high prevalence of MetS and the rising trend of obesity in our population [15,16], we examined the impact of gender-stratified weight change on the risk of incident MetS and its components in participants of the Tehran Lipid and Glucose Study (TLGS) cohort during 3.1 years follow-up.

### Subjects

This study was conducted within the framework of the TLGS, a population based prospective study conducted on residents of district 13 of Tehran, with the aim of determining the prevalence of noncommunicable disease-risk factors and developing a healthy lifestyle to decrease these risk factors [17]. In the TLGS, 15005 people aged 3 years and over, living in district 13 of Tehran were selected by a multistage cluster random sampling method; among them, were 10368 subjects aged ≥ 20 years who participated in phase 1 (cross-sectional phase) from 1999–2001. After this phase, subjects were entered to the two, the cohort and the interventional groups. The cohort group consisted of 6437 subjects aged ≥ 20 years and after excluding those with BMI ≤ 18.5 kg/m^2 ^(n = 339) at baseline, 6098 subjects remained. From these, after further excluding subjects with missing data (n = 112), death events (n = 120) and loss to follow up (n = 2399), there were 3467 subjects with full relevant data who underwent the second examination in phase 2 until September 2005 and entered to the current study (participation rate ≈57%). The main reasons for non responders in the follow up study were either migration (30%) or lack of contribution. The Ethical Committee of The Endocrine Research Center of Shahid Beheshti University (Medical Campus) approved the proposal of this study. Informed written consent was obtained from all subjects.

## Methods

Subjects were interviewed privately, face-to-face, interviews being conducted by trained interviewers using pretested questionnaires. Initially, information on age, smoking habits, subjects' physical activity status, education level and medication use for treatment of diabetes, hypertension and lipid disorders was collected. Subjects with current or past history of smoking were called smokers and those who had never smoked were non smokers. Physical activity was categorized to three groups; vigorous, those who had hard physical activity (leisure time or occupational) at least three times a week; moderate, those who had hard physical activity at least once a week regularly; none, those without any regular hard physical activity. Education was categorized to three groups; Illiterate, under diploma and higher than diploma. Weight was measured, while subjects were minimally clothed without shoes using digital scales and recorded to the nearest 100 g. Height was measured in a standing position, without shoes, using tape stadimeter while shoulders were in a normal position. BMI was calculated as weight in kilograms divided by height in meters squared. Waist circumference (WC) was measured at the umbilical level, using unstretched tape meter, without any pressure to body surface, and was recorded to the nearest 0.1 cm. To avoid inter subjective error; all measurements were taken by the same person. To measure blood pressure (BP), subjects were first made to rest for 15 min, and then a qualified physician measured BP twice, during physical examinations in a seated position after one initial measurement for determining the peak inflation level using a standard mercury sphygmomanometer. There was at least a 30-second interval between these two separate measurements, and thereafter the mean of the two measurements was considered as the participant's blood pressure. A blood sample was taken after 12–14 hour overnight fasting. Blood samples were taken in a sitting position according to the standard protocol and centrifuged within 30–45 min of collection. All blood analyses were done at the TLGS research laboratory on the day of blood collection. Fasting plasma Glucose (FPG) was measured by the enzymatic colorimetric method using glucose oxidize. For lipid measurements, triglyceride kit (Pars Azmoon Inc., Iran) was used. Triglyceride (TG) was assayed using enzymatic colorimetric tests with glycerol phosphate oxidize. HDL cholesterol (HDL-C) was measured after precipitation of the apolipoprotein B containing lipoproteins with phosphotungistic acid. Lipid standard (C.f.a.s., Boehringer Mannheim, Germany; cat. no. 759350) was used to calibrate the selectra 2 auto-analyzer for each day of laboratory analyses. All samples were analyzed when internal quality control met the acceptable criteria. Inter- and intra assay coefficients of variation (CV) for TG were less than 1.6% in the baseline, and less than 3.9% in the follow-up examination, respectively. The equivalent CVs for glucose were less than 2.2% at baseline, and less than 3.3% at follow-up examination, respectively. The method for glucose and lipid measurement was the same at both baseline and follow-up.

### Definition of Terms

The MetS was defined according to the Adult Treatment Panel III (ATPIII) guidelines as the presence of three or more of the following [18]: (1) Abdominal obesity (high WC) as WC > 102 cm for men and > 88 cm for women, (2) High TG level (≥ 1.7 mmol/l); (3) Low HDL-C level (< 1.03 mmol/l in men and < 1.29 mmol/l in women);(4) High BP [systolic blood pressure (SBP) ≥ 130 or diastolic blood pressure (DBP) ≥ 85 mm Hg)]; (5) High FPG concentration (≥ 6.1 mmol/l).

### Weight Change

Percentage of weight change (PWC) during the 3.1 year follow-up was assessed in men and women of the study population separately. PWC was calculated as: [(Weight at phase 2 – Weight at phase1/Weight at phase1) × 100]. Based on the distribution of weight change among both genders, the weight change percent was divided into quintiles, which resulted in a relatively similar number of subjects in each group as follows: Weight loss group [PWC <-1.3% men (n = 294), <-2.5% women (n = 404)]; weight stable (referent group) [PWC -1.3 to 1.3% men (n = 277), -2.5 to 1.2% women (n = 412)]; mild weight gain group [PWC 1.4 to 3.9% men (n = 298), 1.3 to 3.9% women (n = 401)]; moderate weight gain group [PWC 4 to 6.9% men (n = 266), 4 to 7.6% women (n = 391)]; large weight gain group [PWC ≥ 7% men (n = 296), ≥7.7% women (n = 428)].

### Data Analysis

All data were analyzed by SPSS software package (SPSS Inc., Chicago, IL, USA; Version 15). Mean (SD) values or frequency (percentage) of baseline characteristics were expressed. The means and percents of the individual variables were compared between baseline and follow-up groups, by using paired t-test and McNemar test, respectively. General Linear Model was used to estimate means of individual MetS parameters at the 3.1 year follow up examination according to 5 PWC groups adjusted for age, baseline weight, smoking status, physical activity, education level, medication use for treatment of diabetes, hypertension and lipid disorders and the corresponding parameter, at baseline. To elucidate the difference and similarity of the effects of PWC according to gender, subjects were stratified into 2 groups by gender. For MetS and each of its components, relative risk (RR) and the 95% confidence intervals (CI) according to the gender-stratified quintiles of PWC were calculated after adjustments of age, baseline weight, smoking status, physical activity, education level and medication use for treatment of diabetes, hypertension and lipid disorders. The second quintile of PWC, which was called the stable group, was considered as the reference. To examine the RR of developing MetS and its components, logistic models were fitted separately in subject who did not have MetS or its components at baseline, respectively.

## Results

In comparison to the subjects who did not attend the follow-up visit, those who attended had higher values of age (44. vs. 40.8 years), BMI (27. 3 vs. 26.5 kg/m^2^), WC (89.2 vs. 87 cm), SBP (120.8 vs. 118.9 mmHg), DBP (78. 6 vs. 77.5 mmHg), FPG (5.5 vs. 5.4 mmol/l), TG (2 vs. 1.8 mmol/l), (all p values < 0.05). However, there was no difference in HDL-C levels between participants vs. nonparticipants.

The current analysis included 3467 subjects (1431 male and 2036 female, sex ratio M: W = 0.7) with a mean (SD) age of 44.4 (14.2) years. At baseline, the average BMI was 27.3 (4.4) kg/m^2^, normally distributed. Also, 31.9% of the total participants had normal weight (BMI 18.5–24.99 kg/m^2^), 42.5% were overweight (BMI 25–29.99 kg/m^2^) and 25.6% were obese (BMI ≥ 30 kg/m^2^). After a mean 3.1 years of follow-up, range of 9 months to 6 years, the cohort gained a mean weight of 1.9 kg with a range of -28 to +39 kg of weight change, and the prevalence of overweight and obesity increased to 43.3% and 31.3%, respectively (Data not shown). As shown in Table 1 all of the anthropometric and metabolic parameters were higher after follow-up except serum TG levels, HDL-C, SBP and DBP. Medication use for dyslipidemia in males and for hypertension and diabetes in both genders were more prevalent at follow-up. There were more smokers in both genders after follow-up. At baseline, 26% of participants had vigorous and 59.9% had low physical activity. Also, 10.7% of study population was illiterate and 77.7% of those had under diploma education. Among men, we found 394 subjects with MetS at baseline and 484 MetS cases at the follow up; however from the latter, 274 subjects overlapped with baseline cases, with 210 new cases of MetS remained. Among women, we found 856 subjects with MetS at baseline and 969 MetS cases at the follow up, of which 699 subjects overlapped with baseline cases, hence 270 new cases of MetS remained. Therefore, from among the 2217 participants free of MetS at baseline we had 480 (210 men and 270 women) new cases of MetS during 3.1 years follow up. The age-adjusted (Iran census, 2006) incidence of MetS was 20.4% (95% CI, 19.6–21.2) in the total population. Incident MetS was found to be higher in women 23.1% (22.3–24) than in men 18.4% (17.6–19.1), P < 0.001.

**Table 1:** General characteristic of the study participants at the baseline examination and follow-up by gender*

	Men (n = 1431)			Women (n = 2036)		
	
General characteristics	At baseline	At follow-up	P value	At baseline	At follow-up	P value
Age (year)	45.7 ± 14.9	48.9 ± 14.8	< 0.001	43.5 ± 13.6	46.8 ± 13.6	< 0.001
Weight (kg)	75.4 ± 12.3	77.6 ± 12.7	< 0.001	68.8 ± 11.8	70.5 ± 12.0	< 0.001
BMI (kg/m^2^)	26.2 ± 3.8	26.8 ± 3.9	< 0.001	28.1 ± 4.6	29.1 ± 4.8	< 0.001
WC (cm)	89.8 ± 10.8	95.4 ± 10.3	< 0.001	89 ± 12.2	92.8 ± 12.3	< 0.001
FPG (mmol/l)	5.4 ± 1.5	5.6 ± 1.7	< 0.001	5.6 ± 2.1	5.7 ± 2.2	< 0.001
Triglyceride (mmol/l)	2.1 ± 1.4	2 ± 1.3	< 0.001	1.9 ± 1.2	1.8 ± 1.2	< 0.001
HDL-C (mmol/l)	1.0 ± 0.2	0.9 ± 0.2	< 0.001	1.2 ± 0.3	1 ± 0.2	< 0.001
SBP (mmHg)	122.3 ± 19.1	120.2 ± 18.5	< 000.1	119.9 ± 19.4	118.2 ± 20.1	< 0.001
DBP (mmHg)	78.6 ± 11.0	75.7 ± 11.1	< 0.001	78.6 ± 10.3	75.8 ± 10.1	< 0.001
ATPIII MetS, no (%)	394 (27.5)	484 (33.8)	< 0.001	856 (42)	969 (47.6)	< 0.001
Smokers, no (%)	574 (40.4)	628 (44.2)	< 0.001	111 (5.5)	138 (6.8)	< 0.001
Medication for, no (%)						
Dyslipidemia	31 (2.2)	43 (3.0)	< 0.001	111 (5.5)	101 (5.0)	< 0.001
Hypertension	94 (6.6)	95 (6.7)	< 0.001	235 (11.6)	270 (13.3)	< 0.001
Diabetes	39 (2.7)	68 (4.8)	< 0.001	109 (5.4)	158 (7.8)	< 0.001

* Plus, minus represents mean ± standard deviation

BMI: body mass index, WC: waist circumference, FPG: fasting plasma glucose, SBP: systolic blood pressure, DBP: diastolic blood pressure

Smokers are those with current or past history of smoking.

ATPIII defined Mets: metabolic syndrome by the ATPIII definition which is indicated in reference 18.

Table 2 showed means of the MetS parameters by gender-stratified PWC quintiles after adjustment of previously mentioned covariates. In both genders there was a strong linear trend of weight gain and worsening of all MetS parameters, except for the FPG level. Furthermore, the age adjusted incidence (95% CI) of MetS by gender-stratified PWC quintiles among the 2217 participants free of MetS at baseline were as followed; weight loss group, 8.6% (8.0–9.1) in men and 10.2% (9.6–10.8) in women; weight stable group, 20% (16.2–17.7) in men and 12.2% (11.5–12.8) in women; mild weight gain group, 17.1% (16.3–17.8) in men and 23.4% (22.6–24.3) in women; moderate weight gain group, 24.7% (23.8–25.5) in men and 31.6% (30.7–32.5) in women; large weight gain group, 22.7% (21.9–23.5) in men and 30.6% (29.7–31.5) in women.

**Table 2:** Adjusted means of the metabolic physiological parameters by weight change groups after 3 years follow-up*

	Gender-stratified percentage of weight change^†^					
		
Metabolic parameters	Loss group (294 M; 404 W)	Stable group (277 M; 412 W)	Mild gain group (298 M; 401 W)	Moderate gain group (266 M; 391 W)	Large gain group (296 M; 428 W)	P value
Men (n = 1431)						
WC (cm)	90.8 (0.5)	93.7 (0.5)	95.3 (0.5)	96.8 (0.5)	100.5 (0.5)	< 0.001
TG (mmol/l)	1.9 (0.1)	2 (0.1)	2.2 (0.1)	2.3 (0.1)	2.6 (0.1)	< 0.001
FPG (mmol/l)	6.9 (0.2)	6.8 (0.2)	6.9 (0.2)	6.9 (0.2)	6.9 (0.2)	0.7
HDL-C (mmol/l)	0.88 (0.02)	0.85 (0.02)	0.86 (0.02)	0.83 (0.02)	0.080 (0.02)	0.001
SBP (mmHg)	119.8 (1.7)	121.9 (1.8)	123.5 (1.8)	124.6 (1.8)	125.6 (1.8)	< 0.001
DBP (mmHg)	74.4 (1.2)	75.4 (1.2)	76.2 (1.2)	77.3 (1.2)	77.7 (1.2)	< 0.001
Women (n = 2036)						
WC (cm)	87.3 (0.5)	90.9 (0.5)	93.2 (0.5)	94.5 (0.5)	98.5 (0.5)	< 0.001
TG (mmol/l)	1.9 (0.07)	2.1 (0.07)	2.1 (0.07)	2.2 (0.07)	2.4 (0.07)	< 0.001
FPG (mmol/l)	6.6 (0.1)	6.4 (0.1)	6.5 (0.1)	6.5 (0.1)	6.6 (0.1)	0.2
HDL-C (mmol/l)	1.05 (0.02)	1.04 (0.02)	1.01 (0.02)	1.01 (0.02)	0.99 (0.02)	0.003
SBP (mmHg)	118.7 (1.2)	119.9 (1.2)	123 (1.2)	122.9 (1.2)	125.1 (1.2)	< 0.001
DBP (mmHg)	74.9 (0.7)	76.1 (0.7)	77.2 (0.7)	77.2 (0.7)	78.6 (0.7)	< 0.001

* Means were adjusted for age, baseline weight, smoking, physical activity, education level, medication use plus the corresponding variable at baseline; numbers in the parentheses present standard error.

^† ^The gender-stratified percentage of weight change were grouped as follow: weight loss group (<-1.3% men, <-2.5% women); weight stable group (-1.3 to 1.3% men, -2.5 to 1.2% women); mild weight gain group (1.4 to 3.9% men, 1.3 to 3.9% women); moderate weight gain group (4 to 6.9% men, 4 to 7.6% women); large weight gain group (≥ 7% men, ≥ 7.7% women).

BMI: body mass index, WC: waist circumference, TG: triglyceride, FPG: fasting plasma glucose, SBP: systolic blood pressure, DBP: diastolic blood pressure

**Figure 1:**
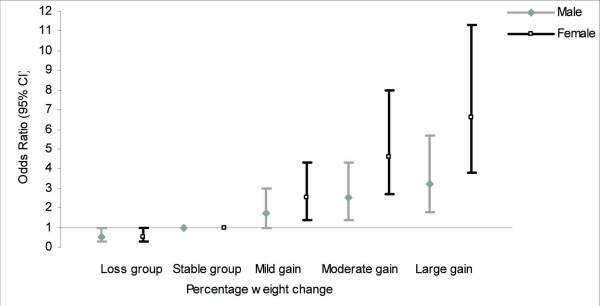
**Adjusted relative risk of metabolic syndrome by weight change groups in both genders**. From the total study population (1431 men and 2036 women), only 1037 men and 1180 women who were free of metabolic syndrome at baseline included in this logistic regression model. The model was adjusted for age, baseline weight, smoking, physical activity, education level and medication use. The gender-stratified percentage of weight change (PWC) was shown in the X axis, grouped as follow: weight loss group [<-1.3% men (n = 191), <-2.5% women (n = 193)]; weight stable group [-1.3 to 1.3% men (n = 185), -2.5 to 1.2% women (n = 204)]; mild weight gain group [1.4 to 3.9% men (n = 209), 1.3 to 3.9% women (n = 228)]; moderate weight gain group [4 to 6.9% men (n = 204), 4 to 7.6% women (n = 233)]; large weight gain group [= 7% men (n = 248), = 7.7% women (n = 322)]. The stable group was considered as reference. Relative risk (95% CI) for ATPIII defined metabolic syndrome according to PWC groups was shown in the Y axis.

Table 3 and Figure 1 showed the adjusted RR (95% CI) of the ATPIII defined MetS and its components according to the weight change groups in men. Mild increase in weight predicted high WC and high TG components, while moderate increase was associated with high WC, high BP components and incident MetS. In this gender, large weight gain predicted MetS and all of its components except high FPG. Weight loss had protective effect on the MetS with the RR 0.5 (95% CI 0.3–1.0) and high WC component with the RR 0.3 (95% CI 0.1–0.6).

**Table 3:** Adjusted relative risk of ATPIII-defined metabolic syndrome components by weight change groups in men (n = 1431)*

	Percentage of weight change				
	
	Loss group (<-1.3%)	Stable group (-1.3 to 1.3%)	Mild gain group (1.4 to 3.9%)	Moderate gain group (4 to 6.9%)	Large gain group (≥ 7%)
High WC	0.3 (0.1–0.6)	Reference	2.4 (1.3–4.4)	4.4 (2.4–8.0)	9.2 (4.8–17.5)
No. of subjects^†^	255	236	263	240	276
P value	0.001		0.005	< 0.001	< 0.001
High TG	0.8 (0.4–1.9)	Reference	2.2 (1.1–4.5)	1.9 (0.9–3.8)	3.8 (1.9–7.7)
No. of subjects^†^	111	103	116	126	168
P value	0.7		0.03	0.08	< 0.001
Low HDL-C	0.8 (0.4–1.4)	Reference	0.9 (0.5–1.6)	1.3 (0.7–2.5)	1.9 (1.0–3.4)
No. of subjects^†^	99	93	108	94	117
P value	0.4		0.8	0.3	0.04
High FPG	0.5 (0.2–1.2)	Reference	1.4 (0.7–2.8)	1.2 (0.6–2.5)	1.3 (0.6–2.9)
No. of subjects^†^	236	234	265	248	281
P value	0.1		0.3	0.6	0.4
High BP	1.2 (0.6–2.7)	Reference	1.8 (0.8–3.7)	2.7 (1.3–5.6)	3.4 (1.7–7.1)
No. of subjects^†^	155	160	175	176	225
P value	0.6		0.1	0.007	0.001

* Relative risks (95% CI) were adjusted for age, baseline weight, smoking, physical activity, education level and medication use.

^† ^Only subjects free of mentioned component at baseline were entered in each analysis.

WC: waist circumference, TG: triglyceride, FPG: fasting plasma glucose, BP: blood pressure

The same analyses in women were shown in Table 4 and Figure 1. In this gender group, increasing weight in any category predicted MetS with the RR ranging from 2.5 (95% CI 1.4–4.3) for mild to 6.6 (95% CI 3.8–11.3) for large weight gain. Mild weight gain showed association with high WC and high BP components and moderate weight gain predicted high WC and high TG components. Large weight gain predicted all components except low HDL-C, on the other hand weight loss found to be protective only against high WC component. Regarding the effect of age on the relationship between weight gain and MetS, especially in women, we divided women participants into those aged > 50 years and ≤ 50 years; the analysis according to this classification highlighted that in women ≤ 50 years the high BP component was not affected by weight gain. However, in those older than 50 years the RRs of high WC and high BP considering weight gain groups, were increased significantly (data not shown).

**Table 4:** Adjusted relative risk of ATPIII-defined metabolic syndrome components by weight change groups in women (n = 2036)*

	Percentage of weight change				
	
Metabolic syndrome components	Loss group (<-2.5%)	Stable group (-2.5 to 1.2%)	Mild gain group (1.3 to 3.9%)	Moderate gain group (4 to 7.6%)	Large gain group (≥ 7.7%)
High WC	0.3 (0.2–0.7)	Reference	1.9 (1.1–3.2)	2.8 (1.6–5.0)	7.3 (4.1–13.0)
No. of subjects ^†^	149	162	206	192	286
P value	.003		.03	< 0.001	< 0.001
High TG	0.7 (0.4–1.2)	Reference	1.2 (0.7–2.1)	2.1 (1.3–3.6)	2.7 (1.7–4.4)
No. of subjects ^†^	173	201	202	204	294
P value	0.2		0.5	0.003	< 0.001
Low HDL-C	0.7 (0.4–1.2)	Reference	0.9 (0.5–1.6)	1.1 (0.6–2.0)	1.5 (0.8–2.7)
No. of subjects ^†^	101	107	96	99	119
P value	0.2		0.7	0.7	0.2
High FPG	0.7 (0.3–1.6)	Reference	1.6 (0.8–3.1)	1.5 (0.7–2.9)	4.3 (2.2–8.2)
No. of subjects ^†^	307	339	340	358	396
P value	0.4		0.1	0.3	< 0.001
High BP	0.9 (0.5–1.8)	Reference	2 (1.1–3.5)	1.3 (0.7–2.5)	2.1 (1.1–3.7)
No. of subjects ^†^	244	240	256	263	338
P value	0.9		0.02	0.3	0.01

* Relative risks (95% CI) were adjusted for age, baseline weight, smoking, physical activity, education level and medication use.

^† ^Only subjects free of mentioned component at baseline were entered in each analysis.

WC: waist circumference, TG: triglyceride, FPG: fasting plasma glucose, BP: blood pressure

As it is possible that using medication could affect the association of weight change and MetS components, we also performed analyses limited to subjects not treated for hypertension, dyslipidemia or diabetes. Results were unchanged after excluding subjects treated with medications (data not shown). Furthermore, when we adapted the American Heart Association/National Heart, Lung, and Blood Institute definition of MetS [19] [which considered medication treatment in MetS components classification] in place of original NCEP/ATP III, the results remained unchanged (data not shown).

## Discussion

In this population based study of Iranian urban residents, we showed that in men, more than a moderate increase in weight (> 4% of the baseline weight) predicted incident MetS, and weight reduction of at least 1.3% of initial body weight plays a protective effect against this syndrome. In women increase in weight, even a mild increase in a short term follow-up (> 1.3% of the initial weight), was associated with an increased risk of incident MetS. In both genders weight loss was protective against high WC component.

There was a 20.6% age-adjusted incident of MetS in our study, which was accompanied by 3.8% incident Type 2 diabetes (FPG ≥ 7 mmol/l or medication use) (data not shown). Onat et al in a representative population of Turkish adults, with a high prevalence of MetS, showed incident cases of MetS in 225 (23%) men and 252 (≈25%) women over a mean follow up of 5.9 years [20,21]. In a 4-year cohort of 9785 Taiwanese adults, there was 17.5% incidence of MetS in men versus 8.3% women, using modified ATPIII definition [22]. Also in non diabetic American Indians, who were free of MetS at baseline, 37.7% got the ATPIII defined syndrome at 10-year follow up [23]. The European Lacidipine Study on Atherosclerosis showed 21.4% incident MetS in hypertensive subjects aged 45–75 years, after 3.7 years follow-up [24].

The effect of weight change on MetS has been proposed to be caused by insulin resistance; it has been also suggested that insulin resistance may lead to MetS through accommodation of adiposity [25]. Weight gain was associated with developing the insulin resistance syndrome in one cross-sectional study on the Finish middle-aged men [8]. The same association of weight gain with incident ATPIII defined MetS was found in the adult American population [26]. Since the aim of this study was descriptive, we did not make large multivariate analyses; so it would have been difficult to interpret the higher incident of MetS in women. However, in a multi center study in different ethnic groups, using signal detection analysis, the best predictor of incident metabolic syndrome was waist circumference [27]. Hence, regarding the high prevalence of abdominal obesity in Iranian females [28]; the higher relative risk for incident metabolic syndrome in this group could be justifiable. The linear association of weight gain with MetS parameters (except FPG) in our study was comparable to the Hiller et al. study, which showed the same association with each of the metabolic physiological continuous measures [6]. Nevertheless, we found a significant decrease in BP, TG and HDL-C during this short term follow up. However, favorable trends in TG and BP were not enough to alleviate the risk of MetS in light of the increasing trend in general and central obesity. Recently, in a non-diabetic population, we showed that despite an increasing trend in general and abdominal obesity, a favorable trend in total cholesterol in both genders and TG in men occurred, which was not related to the increasing usage of lipid lowering drugs. However, this trend was counterbalanced by unfavorable changes in HDL cholesterol, which was found to decrease in both genders especially in men [15]. Similar declining trends in lipoprotein level in light of the increasing trends in obesity were also reported in U.S adult population [29].

Weight loss has been recognized to have beneficial effects on several cardiovascular risk factors [30,31]. Some studies have also shown that weight loss is followed by an improvement of glucose tolerance and a reduced risk of type 2 diabetes [32,33]. Villareal et al. have found that in older obese adults the diet-induced weight loss (≈9% of initial body weight) during 6 months, could improve most of the obesity-related CHD risk factors including high WC [34]. Recently, Alhassan et al. reported that ≥ 7% reduction in body weight has a beneficial impact on MetS variables [35]. In our data analysis, weight loss of > 1.3% of initial weight reduced risk of MetS [RR 0.5, 95%CI (0.3–1.0)] in men; furthermore a mild weight loss decreased risk of the high WC component in both genders.

In our study, weight gain increased the risk of high FPG in women, but not in men. Considering the observational nature of our study, delineating the reason for this divergence between genders regarding weight gain and risk of abnormal glucose metabolism was difficult. However, in Turkish adults with a similar prevalence of MetS, women with normal glucose metabolism were more prone to incident dibetes than men [36]. Furthermore, in a recent population based study from Denmark, women who had gained and sustained considerable weight were more susceptible to development of new onset diabetes [37]. During the short term follow-up in our study, attained BMI (in phase 2) and BMI changes were more marked in women than men (mean of BMI change: 0.99 vs. 0.66 kg/m^2^; P < 0.001, respectively). On the other hand, body weight is a stronger predictor of Type 2 diabetes than physical activity, considering the finding that physical activity was significantly lower in Iranian women than men [38,39]. Hence, the higher prevalence of diabetes and abnormal glucose metabolism (i.e. impaired fasting glucose or impaired glucose tolerance) in Iranian women than in men was justifiable [40].

Overall we also found the association of high BP component with weight gain in both genders. Furthermore, the synergistic contribution of insulin resistance and menopause to components of the metabolic syndrome was reported recently with SBP, DBP and WC [41], this may explain the accelerated impact of weight gain on abdominal obesity and blood pressure among women aged ≥ 50 in the current study. In a cohort study of adult men, an increased in BMI was found to be associated with an increased risk of hypertension and weight loss did not affect risk of hypertension significantly [9].

The results of our study are difficult to compare with other studies conducted on the association of weight change and cardiovascular risk factors because of differences in the time period between the two weight measures, the length of follow up, and how weight change is expressed. There are some other points that should be considered when examining the results of our study. First, about 43% of the baseline participants were excluded from cohort analysis because of loss to follow-up or missing data. This group was healthier in their baseline characteristic assessments; hence we may have overestimated the incidence of metabolic syndrome in the general population. Second, our findings were derived from an observational prospective study; so we did not have information on whether weight loss in this cohort was intentional or unintentional. Finally it must be emphasized that the conclusions drawn from this study were determined in Middle East Caucasian residents in the capital city of Iran and further studies should be conducted to determine whether our findings can apply to other populations of this region.

Among the existing studies addressing the relationship between weight change and MetS, only a few have analyzed their data on the longitudinal effects of weight change adjusting for confounding variables [7,10]. We were also able to adjust for several important confounding variables and to explore the effects of different weight change groups in separate genders. Moreover, we used anthropometric variables that were directly measured by trained medical professionals rather than self-reported or self-measured anthropometrics.

## Conclusion

In this population based cohort study we found a strong positive association of weight gain and increased risk of MetS, especially in women. Furthermore, weight loss had protective effects on the incident MetS in men and high WC component in both genders. Along with previous findings, these data suggest that promoting weight control in the Iranian population is crucial to prevent increasing risk of MetS.

## Competing interests

The authors declare that they have no competing interests.

## Authors' contributions

AZ participated in conception and design of the study, performing the statistical analysis and drafting the manuscript. FH participated in the conception and design of the study, drafting the manuscript, critical revision and its final approval. PS participated in the statistical analysis of the study. FA participated in its design and coordination. All authors read and approved the final manuscript.

## Pre-publication history

The pre-publication history for this paper can be accessed here:


